# 

**Published:** 1894-01

**Authors:** 


### TABLE OF CONTENTS.

ORIGINAL COMMUNICATIONS. PAGE

Etiology of Pyorrhœa Alveolaris. By C. N. Peirce, D.D.S. . . . . . . . 1

The Advantages and Disadvantages of Specialism. By Reuen Thomas, D.D., Ph.D., Brookline, Mass. . . . . . . . . . . . . . . . . . . . . . . 9

Hypnotism, Animal Magnetism, and Personal Magnetism as applied to Dentistry. By W. G. A. Bonwill, D.D.S. . . . . . . . . . . . . . . 21

Pyorrhœa Alveolaris. By Dr. F. T. Van Woert . . . . . . . . . . . . 30

Some Diseases of the Gums: Their Causes and Treatment, with Sketches of Related Incidents in Office Practice. By D. M. Sabater, A.B., D.D.S., M.D., New York . . . . . . . . . . . . . . . . . . . . . . . . . . 34

Incidents connected with the Trial of Professor Webster. By Dr. Lester Noble, Springfield, Mass. . . . . . . . . . . . . . . . . . . . . . 41

REPORTS OF SOCIETY MEETINGS.

New York Odontological Society . . . . . . . . . . . . . . . . . . . 44

Odontological Society of Pennsylvania . . . . . . . . . . . . . . . . 54

Central Dental Association of Northern New Jersey . . . . . . . . . . . 64

EDITORIAL.

The Opening Year . . . . . . . . . . . . . . . . . . . . . . . . . . 70

Diplomas in Absentia still in Demand . . . . . . . . . . . . . . . . . 72

Two Good Papers . . . . . . . . . . . . . . . . . . . . . . . . . . 73

Registering Diplomas in Pennsylvania . . . . . . . . . . . . . . . . . 73

BIBLIOGRAPHY . . . . . . . . . . . . . . . . . . . . . . . . . . . 74

NOTES AND COMMENTS . . . . . . . . . . . . . . . . . . . . . . . 77

CURRENT NEWS.

Dr. Jesse C. Green honored . . . . . . . . . . . . . . . . . . . . . 78

